# Recombinant Human Nerve Growth Factor (Cenegermin)–Driven Corneal Wound Healing Process: An Evidence-Based Analysis

**DOI:** 10.3389/fphar.2021.760507

**Published:** 2022-01-28

**Authors:** Chiara Bonzano, Sara Olivari, Carlo Alberto Cutolo, Angelo Macrì, Daniele Sindaco, Davide Borroni, Elisabetta Bonzano, Carlo Enrico Traverso

**Affiliations:** ^1^ Eye Clinic, Department of Neuroscience, Rehabilitation, Ophthalmology, Genetics, Maternal and Child Health, University of Genoa, Genoa, Italy; ^2^ IRCCS San Martino Polyclinic Hospital, Genoa, Italy; ^3^ Cornea Unit, Royal Liverpool University Hospital, Liverpool, United Kingdom; ^4^ Department of Radiation Oncology, IRCCS San Matteo Polyclinic Foundation, Pavia, Italy; ^5^ PhD School in Experimental Medicine, University of Pavia, Pavia, Italy

**Keywords:** cenegermin, neurotrophic keratoplasty, corneal diseases, ocular pharmacology, pharmacological targets, anterior segment optical coherence tomography (AS-OCT), rh-NGF, autologous serum

## Abstract

**Purpose:** To evaluate anterior segment optical coherence tomography (AS-OCT) to detect the wound healing process as per monitoring the effectiveness of cenegermin to treat moderate to severe neurotrophic keratoplasty.

**Methods:** A retrospective chart review was realized to identify patients treated with cenegermin at the Clinica Oculistica, University of Genoa, Italy. All patients underwent careful examinations at baseline and follow-up visits. AS-OCT scans centered on the minimum corneal thickness (CT) area were always performed. We compared findings of AS-OCT with the findings from the slit-lamp examination. A linear regression analysis was used to evaluate factors associated with corneal healing. A further analysis, including a control group treated with 50% autologous serum (AS), was done to investigate and compare the efficacy of cenegermin.

**Results:** Data from 16 eyes were studied. The average patients' age was 60.9 ± 21.1 years; five (31.2%) eyes experienced persistent epithelial defect and 11 (68.8%) eyes had neurotrophic corneal ulcer. The average reepithelialization time was 3.9 ± 0.5 weeks in the cenegermin group versus 5.9 ± 1.9 weeks in the AS group (*p* < 0.01). The AS‐OCT scans revealed an average CT at the thinnest point of 276.3 ± 74.1 μm before treatment with an average increase of 176.5 ± 60.3 μm at the end of the cenegermin treatment (B = −0.15; *p* = 0.035). The AS-OCT percentage increase in corneal thickness between the two groups was statistically significant (*p* < 0.02).

**Conclusion:** Understanding the cascade of events involved in the nerve growth factor–driven corneal wound healing process is clinically meaningful for the clinician. AS-OCT is an effective tool for systematic anterior segment imaging, allowing the detailed detection of the front-to-back layered corneal structure for quantitative analysis and monitoring of the healing process.

## Introduction

Several ocular and systemic diseases have been associated with damage to the fifth cranial nerve axons, from the trigeminal nucleus to the corneal nerve endings, possibly resulting in the development of neurotrophic keratoplasty (NK) (Dua et al., 2018).

Among the multitude of causes of NK, herpetic keratoplasty (herpes simplex and herpes zoster viral infection) are the most common, followed by intracranial space-occupying lesions or neurosurgical procedures that damage the trigeminal ophthalmic branch (Sacchetti and Lambiase, 2014).

Other ocular causes of NK include chemical burns, physical injuries, corneal dystrophy, chronic use of topical medications (topical anesthetics, timolol, betaxolol, sulfacetamide, and diclofenac sodium), and anterior-segment surgery involving nerve damage (Semeraro et al., 2014). Additional systemic conditions associated with corneal nerve impairment are diabetes mellitus (DM), multiple sclerosis, congenital syndromes, and leprosy (Sacchetti and Lambiase, 2014).

Corneal blindness is the fourth leading cause of blindness worldwide (Jeng and Ahmad, 2021); however, nearly 80% of all cases are avoidable and reversible (Jeng and Ahmad, 2021). Thus, treating corneal diseases such as trauma or keratoplasty represents an urgent or emergent care issue for ophthalmologists. For these reasons, more evidence on new and recent drugs for conservative treatment is highly required, especially in such a crucial time where there is a restriction regarding access to surgical corneal treatments (Toro et al., 2020).

Cenegermin is a novel recombinant human nerve growth factor (rh-NGF) recently approved to treat moderate to severe NK (European Medicines Agency, 2021). Cenegermin eye drop proved to be safe and successful in regenerating corneal integrity in two phase-II clinical trials (Bonini et al., 2018a; Bonini et al., 2018b). The understanding of the cascade of events involved in the NGF-driven corneal wound healing process and the evaluation of how corneal wound healing affects corneal biomechanics and optics are crucial to improving the outcome of the treatment.

Anterior segment optical coherence tomography (AS-OCT) is a noninvasive instrument for systematic anterior segment imaging, allowing the detection of the front-to-back layered corneal structure with sufficient detail to make a quantitative analysis. There have been reports on the assessment of corneal thickness (CT) (Muscat et al., 2002; Ishibazawa et al., 2011); AS-OCT also contributes to the diagnosis of anterior eye diseases, monitoring of pathological conditions, and their healing process (Nubile et al., 2011; Mastropasqua et al., 2017; Venkateswaran et al., 2018; Kowalczyk et al., 2020). OCT technology is also widely used for posterior segment imaging; several studies have shown its effectiveness in disease monitoring with good intra-session reproducibility even in case of corneal pathologies (Reibaldi et al., 2012), as well as in detecting the onset of possible treatment-related complications (Ceravolo et al., 2020; Arumuganathan et al., 2021). Furthermore, it is commonly used as a screening tool for not only ocular diseases but also systemic diseases (Chisari et al., 2019; Carnevali et al., 2021; Koman-Wierdak et al., 2021; Wiest et al., 2021; Zweifel et al., 2021). Recently, AS-OCT has been introduced as a valid tool to optimize the classification of Stage 3 NK (Mastropasqua et al., 2019). The use of AS-OCT for the corneal morphological analysis in patients treated with cenegermin has yet to be described. Our study aims to evaluate AS-OCT to detect the wound healing process as per monitoring the efficacy of cenegermin. A further analysis, including a control group, was performed to investigate and compare the efficacy of cenegermin.

## Materials and Methods

A retrospective chart review was performed to identify patients treated with topical cenegermin eye drops at the Clinica Oculistica, University of Genova, Italy. To evaluate the ability of AS-OCT in detecting the wound healing process as per monitoring the efficacy of cenegermin, the AS-OCT data were compared with an historical control group of patients affected by NK who received standard conventional treatment, 50% autologous serum (AS) eye drops, since 2018 at the same institution.

### Patients Inclusion and Exclusion Criteria

The inclusion criteria were unilateral Stage 2 or 3 NK refractory to one or more conventional treatments for at least 2 weeks, evidence of decreased corneal sensitivity, and the presence of negative corneal cultures, which were taken at admission to our department before the treatment. Finally, only patients with at least 12 months of follow-up after treatment were included.

The diagnosis of NK was established using the Mackie classification: Stage 2 [persistent epithelial defect (PED)] or Stage 3 [neurotrophic corneal ulcer (NCU)]. According to Mastropasqua et al., 2019, the Stage 3 enclosed Stage 3A (corneal ulceration with stromal thinning ≤50% of the total CT) and Stage 3B (corneal ulceration with stromal thinning ≥50% of the total CT). During the treatment period with cenegermin, therapeutic contact lens application and additional topical treatments were discontinued. In addition, possible impending perforation or surgical management needs, and active ocular infection or inflammation unrelated to NK were considered as the exclusion criteria, as were pregnancy and breastfeeding. The same eligibility criteria were applied to enclose the 16 historical controls.

### Data Collection

All patients underwent a complete ophthalmic evaluation before starting the treatment and at each follow-up visit on a weekly basis for 8 weeks. Clinical assessment included slit-lamp (NIKON FS-3V Zoom Photo slit-lamp) examination implemented with corneal epithelial fluorescein staining, epithelialization times weekly monitoring, and AS-OCT (RTVue-100 applied anterior corneal module; Optovue, Fremont, CA). The size (mm) and depth (μm) of NK were recorded. Corneal hypoesthesia was confirmed using a Cochet–Bonnet aesthesiometer. At the first and at each follow-up visit, several AS-OCT scans were performed: pachymetric map, horizontal raster scan centered on the thinnest area of the cornea (17 horizontal scans within a height of 6 mm), cross-line (horizontal and vertical 8.00-mm scans), and line mode (horizontal 8.00-mm scans) on the area of minimum thinning. All scans were centered in the same area at the different visits. We identified the thinnest part of the cornea and measured the corneal and stromal thickness of this portion using the caliper tools that were built into the RTVue-100 system.

### Intervention

In the treatment group, we used a sterile, preservative-free ophthalmic solution containing 0.002% (0.02 mg/ml) of the active ingredient, cenegermin (Oxervate^®^, Dompé Farmaceutici Spa, Milan, Italy). It was packaged in a carton of seven multidose vials (1.0 ml). The patients were instructed to use one vial per day before being discarded and to keep the remaining vials in the carton refrigerated between 2°C and 8°C until the time of use. The scheduled treatment was one drop, six times a day at 2-h intervals for 8 weeks.

In the control group, we used 50% AS eye drops. All patients were screened for blood-borne infections, including syphilis, Hepatitis B and C, and HIV serology before the preparation of AS eye drops. Venipuncture was performed at the antecubital fossa under aseptic conditions to collect 100 ml of whole blood into sterile containers. These were left standing for 2 h to ensure complete clotting. The blood was then centrifuged for 15 min at 3000 RPM. The serum was then separated in a sterile manner and diluted to 50% using saline solution. Patients were recommended to keep the serum at 4°C. We suggested using a bottle for 1 week and administering this six times daily for 8 weeks. Additionally, this group received a prophylactic topical antibiotic (Exocin, Allergan), twice a day. The patients were seen regularly every 7 days for 8 weeks.

### Outcome Measures

• Corneal healing (time frame: at Week 8)

The primary outcome of this study was to achieve complete corneal healing of PED or NCU at Week 8, defined as no corneal fluorescein staining in the area of PED or NCU.

• Quantitative analysis and monitoring of the healing process (time frame: at Week 8)

The secondary end point was to assess the CT, stromal, and epithelial changes through AS-OCT (RTVue-100) and to register any adverse reactions to drug or any disease recurrence with at least 12 months of follow-up.

### Statistical Analysis

We compared the results of the AS-OCT scans with the biomicroscopic findings. The variables were summarized with mean and standard deviation for continuous variables and absolute value and percentage for frequency of categorical variables. In addition, a univariate linear regression analysis was performed to identify variables associated with the percentage change of CT by AS-OCT at the thinnest point. A standard statistical two-tailed paired *t* test was used to estimate the statistical significance of the differences between groups. All statistical analyses were performed with Stata version 15.1 (StataCorp LP, College Station, TX). The alpha level (type I error) was set at 0.05 for all analysis.

## Results

### Affected Eyes in the Cenegermin Group

There were 11 female and five male patients with a mean age of 60.9 ± 21.1 years (range, 7–88 years) in the cenegermin group. In this group, five (31.2%) eyes experienced PED and 11 (68.8%) eyes had NCUs to varying degrees. According to the substages of NK, at Stage 3 of the Mackie's classification suggested by Mastropasqua et al. (2017), seven were Stage 3A and four were Stage 3B.

Affected eyes with PED: one recurrent herpetic epithelial lesion despite antiviral therapy, one epithelial defect after surgical treatment for acoustic nerve neuroma refractory to previously tarsorrhaphy, one PED developed on a previously transplanted cornea, one PED occurred in a child affected by a diffuse midline glioma, and one developed in the context of severe dry eye concomitant to corneal anesthesia.

Affected eyes with NCU: a cornea ulcer subsequent to a severe alkali burn, three recurrent herpetic stromal ulcers, and one NCU developed on a previously transplanted cornea in a diabetic patient. Four NCU occurred in eyes previously underwent surgical retinal detachment repair (Pars Plana Vitrectomy and endolaser). One NCU developed in the context of systemic polyneuropathy, and one occurred after a long-term topical glaucoma medication concomitant to corneal hypoesthesia.

**FIGURE 1:**
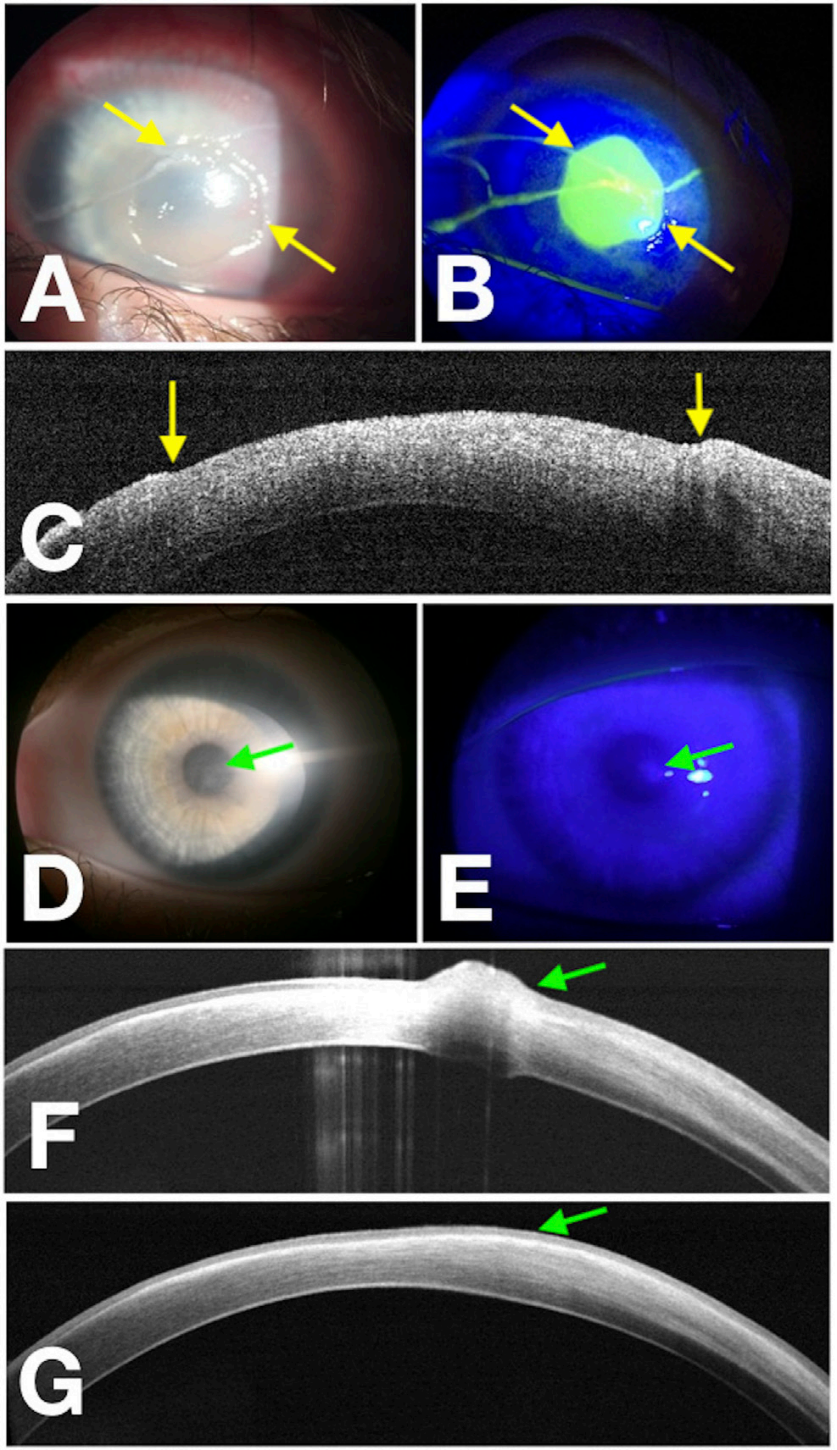
Corneal wound healing of pediatric NK (rh-NGF Case 1) imaged with a multimodal approach. Yellow arrows point at the edges of the epithelial fronts in the photograph obtained at baseline with diffuse white light **(A)**, with fluorescein staining (green) photograph obtained under cobalt-blue light illumination **(B)**, and points to the epithelial edges in the OCT scan over the thinnest area at baseline **(C)**. **(D–F)** Images acquired at the end of the treatment at Week 8, and the green arrows show a residual paracentral corneal epithelial hyperplasia. About 1 month later, OCT scan **(G)** reveals complete regression of the corneal epithelial hyperplasia.

**FIGURE 2:**
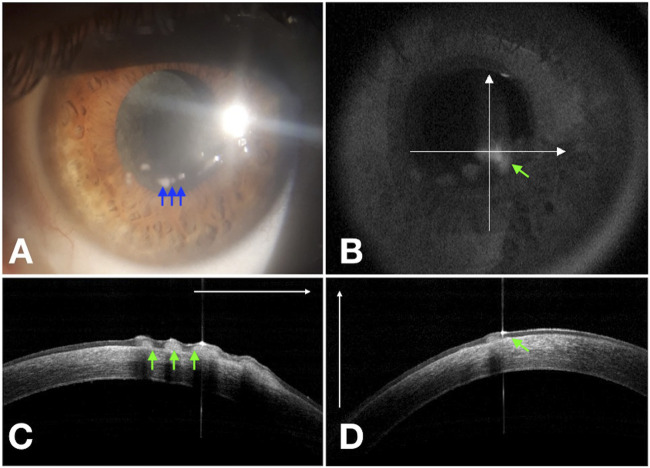
Residual corneal epithelial hyperplasia (blue arrows) of NK (rh-NGF Case 4) at Week 8 **(A)**. Cross-line OCT **(B)** confirms the epithelial hyperplasia (green arrows) in the horizontal **(C)** and vertical **(D)** scans.

All patients were treated by cenegermin eye drops six times a day. A total of 16 eyes of 16 patients healed completely with minimal scarring. There was no recurrence in these patients during the 12 months of follow-up time. Three eyes affected by PED (60% of PED) experienced epithelial hyperplasia regressed within the following month (Figure 1 and Figure 2). The demographic data, etiologies, stages of NK before the treatment, and percentage increase in CT after cenegermin administration are summarized in Table 1.

**TABLE 1:** Ocular and demographic characteristics of the treated cases and the control group, *measured by AS-OCT.

	N	Age	Gender	Eye	Underlying cause	NK (stage)	Healing time (weeks)	Post-TP CT* (%)
**rh-NGF**								
						**PED**		
	1	7	M	OS	Neurosurgical procedure	2	3	73.2
	2	47	M	OD	Neurosurgical procedure	2	4	31.9
	3	88	F	OD	Herpetic eye disease	2	3	20.6
	4	46	F	OD	Corneal hypoesthesia	2	4	54.8
	5	74	F	OS	keratoplasty	2	4	50
*mean ± SD*		*52.4 ± 31.1*					*3.6 ± 0.5*	*46.1 ± 20.5*
						**NCU**		
	6	76	F	OD	DM/keratoplasty	3 (IIIa)	5	53
	7	64	M	OD	Herpetic eye disease	3 (IIIa)	3	81.5
	8	76	F	OS	Herpetic eye disease	3 (IIIa)	4	73.2
	9	58	F	OS	Ocular surgery	3 (IIIa)	4	69.5
	10	45	M	OD	Ocular surgery	3 (IIIa)	5	96.2
	11	86	F	OS	Polyneuropathy	3 (IIIa)	3	62
	12	39	M	OD	Ocular surface injury (Chemical burn)	3 (IIIa)	4	65.3
	13	61	F	OD	Ocular surgery	3 (IIIb)	4	85
	14	53	F	OD	Ocular surgery	3 (IIIb)	4	90.4
	15	79	F	OS	Glaucoma medication (drops)	3 (IIIb)	4	85.6
	16	75	F	OS	Herpetic eye disease	3 (IIIb)	4	74
*mean ± SD*		*64.7 ± 15*					*4 ± 0.5*	*75.9 ± 13.1*
**PED + NCU**		***60.9 ±21.1*** (***7–88***)					***3.9 ±0.5***	***66.6 ±20.7***
**AS**								
						**PED**		
	1	81	M	OS	Herpetic eye disease	2	8	14
	2	68	F	OD	Corneal hypoesthesia	2	7	10
	3	70	F	OD	Herpetic eye disease	2	7	13.9
	4	61	F	OD	Glaucoma medication (drops)	2	3	9.5
	5	80	F	OS	Herpetic eye disease	2	5	9.1
*mean ± SD*		*72 ± 8.5*					*6* ***±*** *2*	*11,3* ***±*** *2.4*
						**NCU**		
	6	69	F	OD	Severe dry eye	3 (IIIa)	3	12.4
	7	77	F	OS	Severe dry eye	3 (IIIa)	4	15
	8	86	M	OS	Ocular surgery	3 (IIIa)	8	16
	9	74	F	OS	Herpetic eye disease	3 (IIIa)	8	18
	10	84	F	OD	Ocular surgery	3 (IIIa)	8	17
	11	47	M	OD	Neurosurgical procedure	3 (IIIa)	7	23.9
	12	53	F	OS	Polyneuropathy	3 (IIIa)	6	11
	13	56	M	OS	keratoplasty	3 (IIIa)	4	19
	14	77	F	OD	Severe dry eye	3 (IIIa)	5	16
	15	13	M	OS	Ocular surgery	3 (IIIb)	3	18
	16	79	F	OD	Neurosurgical procedure	3 (IIIb)	8	13
*mean ± SD*		*65 ± 21.5*					*5.8* ***±*** *2.1*	*16.3* ***±*** *3.6*
**PED + NCU**		***67.2 ±18.4*** (***13–86***)					***5.9 ±2***	***14.7 ±4***

n=number, SD=standard deviation, PT CT* (%)= post-treatment percentage increase in corneal thickness

### Affected Eyes in the Autologous Serum Group

In the control group, there were 11 female and five male patients, and the mean age was 67.2 ± 18.4 years (range, 13–86 years). There were five (31.2%) PED and 11 (68.8%) NCU, with only two in Stage 3B.

Affected eyes with PED: three recurrent herpetic epithelial lesions, one PED developed in the context of corneal hypoesthesia, and one occurred after a long-term topical glaucoma medication.

Affected eyes with NCU: one recurrent herpetic stromal ulcer, one NCU developed on a previously transplanted cornea, and one NCU refractory to previously amniotic membrane transplantation. Two NCU occurred in eyes that had previously undergone surgical retinal detachment repair (Pars Plana Vitrectomy and endolaser) and two NCU resulting from post-surgical trigeminal nerve injury. One NCU developed in systemic polyneuropathy, and three NCU developed in the context of severe dry eye disease.

We administered 50% AS eye drops six times a day. A total of 16 eyes of 16 patients healed completely with minimal scarring. The follow-up time was 12 months, and the defect recurred after the suspension of therapy in four (25%) patients during the follow-up period. The demographic data, etiologies, stages of NK, and percentage increase in CT in the AS control group are summarized in Table 1.

As in the literature data (Bonini et al., 2018a; Bonini et al., 2018b), the most common cause of NK in our patients was herpes simplex keratoplasty, followed by ocular surgery, neurosurgical trigeminal damage, and severe dry eye disease (Table 2).

**TABLE 2:** Participant baseline characteristics demonstrating that there were no significant differences in patient demographics between the treatment and historical control groups.

Baseline characteristics	rh-NGF (*n* = 16 eyes)	As (*n* = 16 eyes)	*p* Value
**Age** (years), mean ± SD (range)	60.9 ± 21.1 (7–88)	67.2 ± 18.4 (13–86)	0.13
**Sex**			0.67
Male	5 (31.2%)	5 (31.2%)	
Female	11 (68.8%)	11 (68.8%)	
**Diagnosis**			
Neurosurgical procedure	2	2	
Ocular surgery	4	3	
keratoplasty	2	1	
Herpetic eye disease	4	4	
Corneal hypoesthesia	1	1	
Ocular surface injury (chemical burn)	1	—	
Glaucoma medication (drops)	1	1	
Polyneuropathy	1	1	
Severe dry eye	—	3	

n = number, SD = standard deviation.

Additionally, three patients reported associated systemic conditions: DM, systemic polyneuropathy, and diffuse midline glioma, respectively.

### Slit-Lamp Monitoring of the Cornea Healing Process

The epithelialization was screened by fluorescein staining test on slit-lamp examination. The mean time for closure of the corneal lesion was 3.9 weeks ± 0.5 (range: 3–5 weeks) and 5.9 weeks ± 1.9 (range: 3–8 weeks) for the cenegermin group and AS group, respectively. The difference in epithelialization times between the two groups was statistically significant (*p* < 0.01).

Then in the cenegermin group, we clinically observed that the cornea healing was faster in patients younger than 75 years, but it was not statistically significant (*p* > 0.05).

In the AS control group, we observed a 25% recurrence rate compared with no recurrence in the cenegermin group.

**FIGURE 3:**
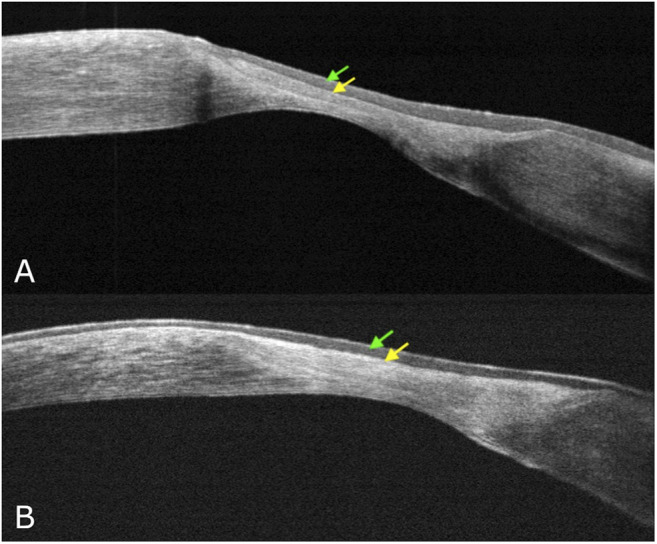
Corneal wound healing AS-OCT detail. First corneal epithelial hypertrophy filled the thinning area, then scar tissue formed **(A)**, and the corneal stroma thickness gradually improved **(B)** (green arrow points to corneal epithelium; yellow arrow points to corneal stroma).

**FIGURE 4:**
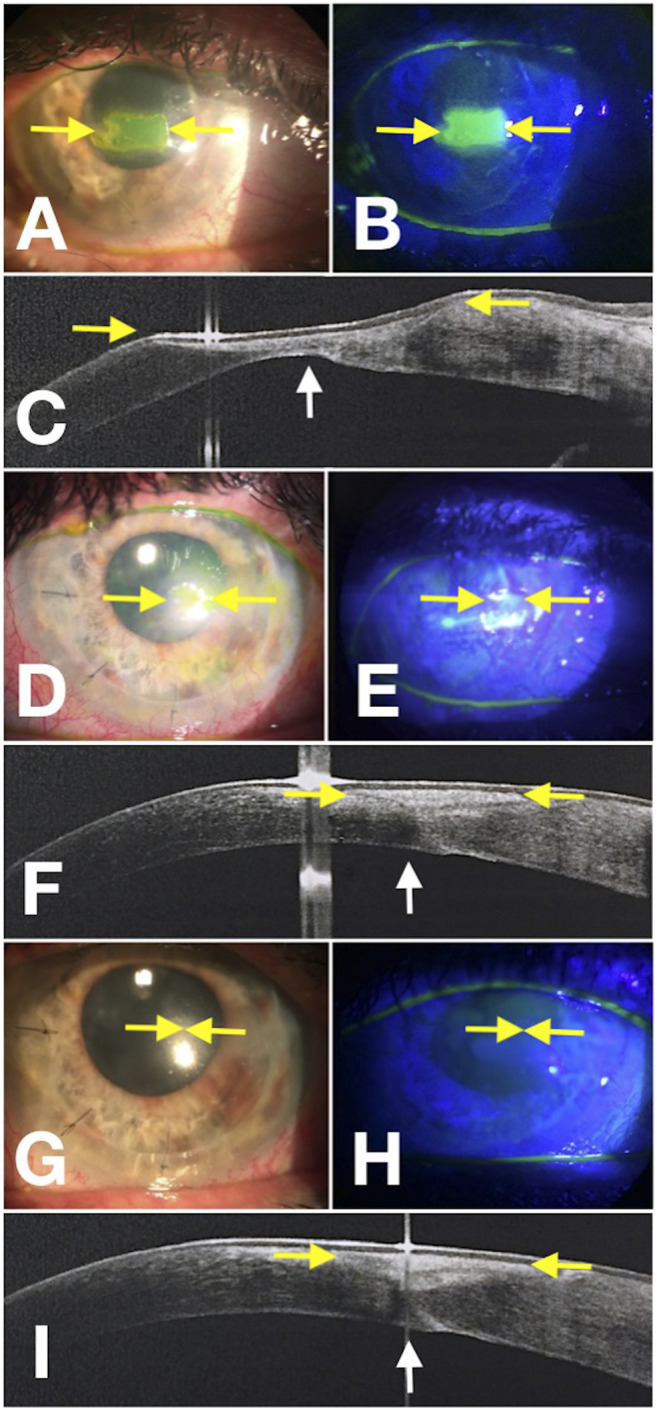
Corneal wound healing of neurotrophic corneal ulcer (rh-NGF Case 6) imaged with a multimodal approach. Yellow arrows point at the edges of the epithelial fronts in the photograph obtained at baseline with diffuse white light **(A)**, with fluorescein staining (green) photograph obtained under cobalt-blue light illumination **(B)**, and points to the ulcer area in the OCT scan detected over the thinnest area at baseline **(C)**. **(D–F)** Images show the progression at Week 4, and the yellow arrows point to the epithelial edges. **(G–I)** Images were acquired at the end of the treatment at Week 8. The white arrows point to the change in CT detected by the AS-OCT scan **(C,F,I)**.

During the first phase of NK healing, opaque slit-lamp scar tissue with high reflectivity on AS-OCT, looking different from the normal corneal stroma, was detected (Figure 3). At first, corneal epithelial hypertrophy filled the thinning area. Then scar tissue formed, and the corneal stroma thickness gradually improved. Second, when the scar tissue disappeared on slit-lamp examination, AS-OCT still detected an increased reflectivity of the anterior stroma at the corneal lesion site (Figure 4). In three PED, epithelial hyperplasia co-occurred with reepithelialization. It was appreciable with both the slit-lamp (Figure 1D,E and Figure2A) and AS-OCT (Figure 1F and Figure 2C). Finally, within a month, it resolved in all three cases and was no longer detectable (Figure 1G).

### AS-OCT Analysis and Monitoring of the Cornea Healing Process

**GRAPH 1:**
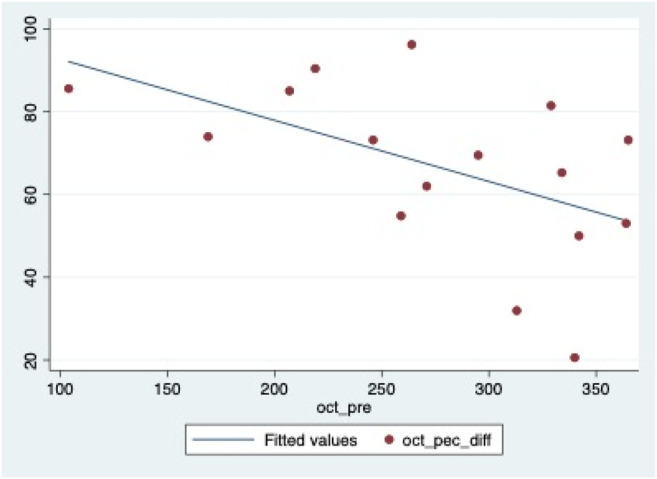
Scattergram representing the association between pre-treatment CT and gain in CT at the end of the treatment.

AS-OCT scans revealed an average CT in the thinnest point of 276.3 ± 74.1 μm at baseline and an average increase in CT of 176.5 ± 60.3 μm at the end of the cenegermin treatment (Week 8). Linear regression showed that the percentage increase of CT at the end of treatment with cenegermin was associated with the pre-treatment CT (B = −0.15; *p* = 0.035) (Graph 1). Table 3 shows the results of the regression analysis. No other variables such as demographic factors or the epithelial defect area were associated with changes detected by AS-OCT.

**TABLE 3:** Linear regression analysis. Outcome: percentage change in CT at the end of cenegermin treatment.

	Coefficient	95% CI	*p*-value
Age, years	−0.35	−0.88 to 0.18	0.179
Gender, female	−9.35	−33.60 to 14.90	0.422
Healing time, weeks	4.88	−14.12 to 23.87	0.591
Area pre-treatment, mm^2^	0.04	−0.15 to 0.24	0.631
CT pre-treatment, μm	−0.15	−0.28 to −0.02	0.035

**GRAPH 2:**
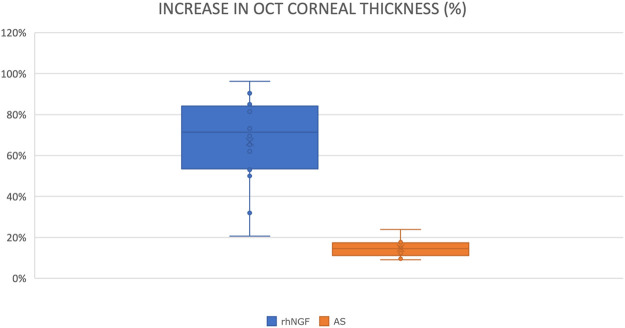
Box plot representing the difference of percentage AS-OCT increase in corneal thickness between the cenegermin (rh-NGF) group and the AS group.

**GRAPH 3:**
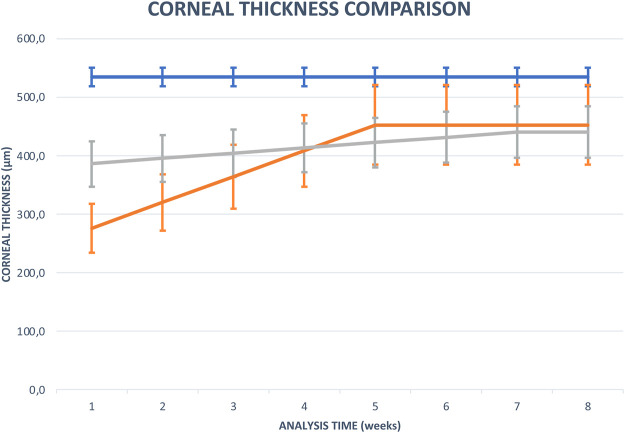
Comparison chart of the mean CT at each time point during the treatment period (8 weeks) between the cenegermin group (orange line), the AS group (gray line), and 16 healthy corneas (blue line).

Compared with the control group, AS-OCT data showed that the average percent change in CT detection was 66.6% [95% confidence interval (CI), 20.6–96.2] in the cenegermin treatment group and 14.7% (95% CI, 9.1–23.9) in the control group. The difference of percentage AS-OCT increase in cornea thickness between the two groups was statistically significant (*p* < 0.02) (Graph 2 and Graph 3).

No side effects were observed in patients who received cenegermin or AS eye drops. Furthermore, there were no serious complications such as infectious keratoplasty that were encountered during the entire study period.

## Discussion

Of our cohort of patients, herpetic keratoplasty was the leading cause of corneal innervation impairment. Neurological and ocular surgical procedures, DM, and chronic glaucoma medication were other conditions involved in the disease pathogenesis.

We showed that baseline CT significantly correlates with post-treatment CT increase. The lower the baseline stromal thickness, the more significant was the effect of cenegermin in restoring CT.

Keratocytes, collagen fibrils, and proteoglycans are essential components of the corneal stroma (Hassell and Birk, 2010). Corneal transparency is guaranteed thanks to the uniform distribution of collagen lamellae (Maurice, 1957; Meek, 2009). This primary ultrastructure could be compromised during the wound healing process, giving way to a progressive swelling and a subsequent corneal opacity, identified as a scar on slit-lamp examination. It means decreased visual acuity in clinical practice, and AS-OCT could detect it as hyper-reflective scar tissue (McCally et al., 2007; Kamma-Lorger et al., 2009; Alafaleq et al., 2021). Type 3 collagen expression is initially significantly improved during the corneal healing process (Cintron et al., 1988). It is synthesized and deposited primarily by fibroblasts; however, it is easily broken down during tissue remodeling (Wilson et al., 2012). The scar tissue formation is probably due to the fibrotic cellular responses and an abnormally large fibril diameter formation (Wilson et al., 2012). First, the scar tissue appeared opaque, as seen with the slit lamp, and had a high signal on AS-OCT, looking different from the normal corneal stroma. Second, the scar tissue disappeared on slit-lamp examination, suggesting that the cornea healing process had switched from an acute wound healing stage to a remodeling one. Utsunomiya et al. (2014) had stated that a long-term AS-OCT hyper-reflectivity persists even when the cornea clears up on slit-lamp examination, and they supposed it could be due to the scar tissue's incapacity to come back to the original arrangement.

The characteristic of scar tissue is an abnormal alignment of the collagen fibrils, directly associated with the tissue's transparency (Zhou et al., 2017). In our case series, we also observed that the younger the patient was, the faster the scar tissue formation, and the less time-consuming the process was to restore transparency. It is only a clinical remark, and there is no statistical significance for the small number of patients. It could mean an age-related cornea healing capacity, confirming age-related changes in the human cornea described in the literature (Marr, 1967; Alvarado et al., 1983; Faragher et al., 1997; Berlau et al., 2002; Roszkowska et al., 2004; Niederer et al., 2007; Gipson, 2013; Bonzano et al., 2021). Mechanisms underlying an age-related slowdown of collagen synthesis in damaged stroma have not been fully delineated yet.

Both cenegermin and AS eye drops proved to be effective in treating NK from different etiologies. Thanks to its biomechanical and biochemical properties, similar to natural tears (Geerling and Hartwig, 2002), the serum has gained wide acceptance in treating ocular surface disorders unresponsive to conventional medical treatment (Tsubota et al., 1999; Matsumoto et al., 2004; Bonzano et al., 2018). Epithelial growth factors such as vitamin A, fibronectin, epidermal growth factor, transforming growth factor b, substance P, and insulin-like growth factor-1 in serum could explain its efficacy in healing cornea lesion, which is usually associated with an already compromised ocular surface (Matsumoto et al., 2004).

Cenegermin in our patients proved to heal the NK faster than the AS and prevent NK recurrence in the 12-months follow-up. According to literature data, this could be explained thanks to the ability of cenegermin in inducing corneal recovery by restoring sensory nerve supply (Bonini et al., 2018b; Pflugfelder et al., 2020).

Both treatments proved to heal NK within 8 weeks in our patients, but with different mechanisms: AS seems to restore the ocular surface providing some neural healers when administered (Matsumoto et al., 2004) and, in four cases, it required to be continued over time to maintain healing; cenegermin, by addressing the underlying cause, induces recovery of corneal nerves, and then it seems to ensure a stable framework even when discontinued with less risk of recurrence (Sacchetti et al., 2021). As recently reported by Pflugfelder et al. (2020), variables such as disease stage, time since diagnosis, and underlying etiologies did not significantly affect the healing status. Furthermore, in our patients, as reported in the literature, no initial NK lesion size (Pflugfelder et al., 2020) impacted significantly; conversely, the pre-treatment cornea thickness detected by AS-OCT was associated with the percentage increase of CT. Therefore, monitoring the healing improvements in terms of thickness and transparency of the cornea by AS-OCT could help understand the corneal healing response to cenegermin.

We used RTVue-100 for AS-OCT that is a spectral-domain OCT that was developed for the retinal analysis. This instrument uses an 840-nm wavelength beam. We are aware that other specialized AS-OCTs by using a 1310-nm wavelength could be more performing (Georgeon et al., 2021). Anyway, RTVue-100 allowed us to detect detailed scans of the corneal healing process and its improvements over time. Thanks to its dedicated caliper tool, it also allowed us to accurately document changes by measuring the stromal thickness at the thinnest part. AS-OCT is an effective tool for systematic anterior-segment imaging, allowing the detailed detection of the front-to-back layered corneal structure for quantitative analysis and monitoring of the healing process (David et al., 2021). Using AS-OCT granted us quantitative monitoring of structural changes in NK treated by cenegermin, obtaining a better understanding of rh-NGF–driven corneal wound healing process.

## Data Availability

The raw data supporting the conclusion of this article will be made available by the authors, without undue reservation.
